# Evaluation of Melongosides as Potential Inhibitors of NS2B-NS3 Activator-Protease of Dengue Virus (Serotype 2) by Using Molecular Docking and Dynamics Simulation Approach

**DOI:** 10.1155/2022/7111786

**Published:** 2022-08-23

**Authors:** Partha Biswas, Ommay Hany Rumi, Dhrubo Ahmed Khan, Md Nasir Ahmed, Nusratun Nahar, Rownak Jahan, Md Nazmul Hasan Zilani, Tridib K Paul, Anamul Hasan, Tohmina Afroze Bondhon, Khoshnur Jannat, Md Nazmul Hasan, Mohammed Rahmatullah

**Affiliations:** ^1^Department of Genetic Engineering and Biotechnology, Faculty of Biological Science and Technology, Jashore University of Science and Technology, Jashore-7408, Bangladesh; ^2^ABEx Bio-Research Center, East Azampur, Dhaka-1230, Bangladesh; ^3^Department of Biotechnology & Genetic Engineering, University of Development Alternative, Lalmatia, Dhaka-1207, Bangladesh; ^4^Department of Pharmacy, Southeast University, Banani, Dhaka-1213, Bangladesh; ^5^Department of Pharmacy, Faculty of Biological Science and Technology, Jashore University of Science and Technology, Jashore-7408, Bangladesh; ^6^Laboratory of Pharmaceutical Biotechnology and Bioinformatics, Department of Genetic Engineering and Biotechnology, Jashore University of Science and Technology, Jashore-7408, Bangladesh

## Abstract

Dengue is a *Flavivirus* infection transmitted through mosquitoes of the *Aedes* genus, which is known to occur in over 100 countries of the world. Dengue has no available drugs for treatment; CYD-TDV is the only vaccine thus far approved for use by a few countries in the world. In the absence of drugs and a widely approved vaccine, attention has been focused on plant-derived compounds to the discovery of a potential therapeutic for DENV. The present study aimed to determine, *in silico*, the binding energies of the steroidal saponins, melongosides, to NS2B-NS3 activator protease of DENV-2, which plays an essential role in the viral replication. The blind molecular docking studies carried out gave binding energies (ΔG = −kcal/mol) of melongosides B, F, G, H, N, O, and P as 7.7, 8.2, 7.6, 7.8, 8.3, 8.0, and 8.0, respectively. All the melongosides interacted with the NS3 protease part of NS2B-NS3. Melongosides B, F, and N showed interactions with His51, while melongoside G interacted with Asp75 of NS3, to be noted, these are important amino acid residues in the catalytic site of the NS3 protease. However, the 200 ns molecular dynamic simulation experiment indicates significant stability of the protein-ligand interactions with the RMSD values of 2.5 Å, thus suggesting a better docking position and no disruption of the protein-ligand structure. Taken together, melongosides need further attention for more scientific studies as a DENV inhibitory agent, which if proven, *in vivo* and in clinical trials, can be a useful therapeutic agent against at least DENV-2.

## 1. Introduction

Dengue viruses belong to the family *Flaviviridae* and consist of four major serotypes, namely, dengue virus 1–4 (DENV 1–4), which are responsible for dengue fever [1]. A fifth serotype (DENV 5) has been reported in 2015, which follows the sylvatic cycle rather than the human cycle followed by the other DENV serotypes [2]. Dengue is a mosquito-borne viral infection which spreads when the virus is transmitted from humans to humans by mosquitoes belonging to the *Aedes* genera, like *Aedes aegypti* and *Aedes albopictus* [3].

Dengue fever in humans is characterized by fever, myalgia (muscle pain), arthralgia (joint pain), abdominal pain, rash, and thrombocytopenia (low counts of platelets). The more severe form of dengue causes dengue hemorrhagic fever, which can result in death [3]. As per the World Health Organization (WHO) fact sheet of 19 May 2021, the global incidence of dengue has grown in recent decades; about half the world's population is now at risk from dengue, and there are an estimated 100–400 million infected cases per year. Furthermore, there is no drug against the virus, and a single vaccine Dengvaxia® (CYD-TDV) developed by Sanofi Pasteur was licensed in December 2015 and has now been approved by the regulatory authorities in only ∼20 countries [4].

The dengue virus carries a positive single strand RNA in its genome. The viral genome is encoded by three structural and seven nonstructural proteins (NSPs) [5]. Of the NSPs, NS2B/NS3 protease is considered to be an excellent therapeutic target for inhibiting the dengue virus because a serine protease domain present in the NS3 plays an integral role in the viral replication [6–8]. As a prime therapeutic target, the NS2B/NS3 has been widely studied for inhibition with natural compounds like panduratin, synthetic compounds like benzimidazole, and *in silico* methods, utilizing mostly the molecular docking programs [9, 10]. *In silico* studies, in turn, have screened various phytochemicals for their binding affinity to various nonstructural proteins of DENV [11].

Plant-derived saponins, such as triterpene saponins [12, 13] and glycosteroidal saponins [14, 15], have been reported to exert the antiviral activity. Since plant-derived saponins have been reported for antiviral activities against both RNA [16] and DNA viruses [17], 3 steroidal saponins that were isolated from the roots of *Solanum sisymbriifolium* Lam. have shown significant antiviral effects against DENV-2 with EC_50_ values ranging from 24.9 to 35.1 *μ*g/mL [18]; the present study aims to evaluate the binding energies of the steroidal saponins, melongosides, to NS2B-NS3 activator protease of DENV-2 via *in-silico* approaches by using the molecular docking and molecular simulations techniques.

## 2. Materials and Methods

### 2.1. Receptors and Ligand Selection

For this study, several bioactive phytochemicals with potential medicinal effects were considered, named as melongoside B, melongoside F, melongoside G, melongoside H, melongoside N, melongoside O, and melongoside P. The NS2B-NS3 protease of Dengue 2 (DENV-2) [19], PDB ID-2FOM, has been selected as the targeted receptor and the established inhibitor of DENV-2, and quercetin was considered as the standard reference for the study [20].

### 2.2. Protein Preparation and Generation of Receptor Grids

The NS2B-NS3 protease of Dengue 2 (DENV-2), PDB ID-2FOM was used as the targeted receptor protein for this study. The RCSB PDB Protein Data Bank (https://www.rcsb.org/) has been accessed to find the structure of the protein in the PDB and the Protein Preparation Wizard of Maestro Desmond version 12.5 (Schrödinger Release 2020-3 Schrödinger, LLC, New York, NY, 2020) was used to prepare the protein for molecular docking application. In order to achieve the desired results, the following criteria were used: assign bond orders, use the CCD database, add hydrogens, create zero-order bonds to metals, create disulfide bonds, fill missing side chains and loops by using prime, fixed cap termini, and delete waters beyond 5 from heat groups; and generate heat states of pH 7.0 ± 2.0 using Epik. The results were evaluated using the following criteria: assign bond orders, use the CCD database, add hydrogens, and generate zero. Using the refine tab and the OPLS3e force field, the H-bond was assigned to PROPKA pH level 7.0, as well as the devaluation, was constrained to RMAD 0.30 using the converging heavy atom. After that, the receptor grid was generated by targeting the natural ligand of the protein.

### 2.3. Ligand Compounds' Preparation

All the three-dimensional (3D) structures of the phytochemicals and the standard were obtained in SDF format from the open-source PubChem database (https://pubchem.ncbi.nlm.nih.gov/) using the structure data format (SDF). All ligand structures were constructed for molecular docking using the LigPrep. To make things easier, we used the OPLS3e force field to make things smaller and the Epik Ionizer to make them smaller. The maximum number of conformers per structure was 32 and the RMSD for each structure was 1.0 Å.

### 2.4. Molecular Docking and Visualization

The extra precision (XP) type of molecular docking was conducted by using the Maestro (Schrödinger Release 2021-2: Maestro, Schrödinger, LLC, New York, NY, 2020-3) and the PDB structure of all protein-ligands complexes was retrieved for post-docking analysis. Ligplot + version 2.2 was used to analyze the noncovalent (polar and hydrophobic) interactions between the protein-ligand complexes. This visualization tool performed admirably because of the Java interface (Java SE Runtime Environment 8u271), which permitted only the combined PDB files created with the PYMOL tool [21]. Moreover, for the validation of complexes' structural bonding compactness, polar and nonpolar interaction bonds, Discovery Studio Visualizer (https://media.accelrys.com/downloads/visualizer/45/DS45Client.exe) 64 bit was run.

### 2.5. Molecular Dynamic Simulation

The 200 ns MD simulations analyzed the protein-ligand complex structures to determine the binding consistency of the selected three candidate ligand compounds to the targeted protein NS2B-NS3 protease of Dengue 2 (DENV-2), PDB ID-2FOM [22]. The molecular dynamic simulation of the protein-ligand complex structures was carried out by using the “Desmond v3.6 Program” in Schrödinger (https://www.schrodinger.com/) (paid version) in a Linux environment to analyze the thermodynamic stability of receptor-ligand complexes [23]. A predetermined TIP3P (transferable intermolecular potential with 3 points) water technique was developed for this framework to maintain a specified volume with an orthorhombic periodic bounding box shape separated by a distance of 10 Å. For electricity neutralization in the framework, appropriate ions such as 0+ and 0 and 15 M salt have been selected and randomly distributed throughout the solvent system. Following the construction of the solvency protein systems with a ligand complex, the system framework was lowered and relaxed by employing the standard procedure performed by using force field constants OPLS3e within the Desmond package [22]. All NPT assemblies that made use of the temperature's combination of the Nose–Hoover thermostat and the isotropic approach were maintained at 300 K and at one-atmosphere pressure (1,01325 bar) and were accompanied by 50 PS capturing intervals with an efficiency of 1.2 kcal/mol. The simulation's entire event was evaluated using the Simulations Interaction Diagram (SID) of the Desmond modules included in the Schrödinger suite that was performed to evaluate the MD simulation's accuracy. The stability of the protein-ligand complex system was determined using the root-mean-square deviation (RMSD), protein-ligand contacts (P-L), intramolecular hydrogen bonds, solvent accessible surface area (SASA) value, radius of gyration (Rg) value, MolSA, and the polar surface area (PSA) values.

## 3. Results

### 3.1. Interpretation of Molecular Docking

A molecular docking technique using the Maestro package platform was used to identify phytochemicals that interact with the NS2B-NS3 protease of the Dengue 2 (DENV-2) protein. The Maestro application produced the greatest possible docking score for macromolecules and ligands. Quercetin (control) was used as a control ligand in this investigation and it had a binding affinity of −8.2 Kcal/mol.  Table 1 shows that the ligand melongoside P had the best fitting score of −9.5 Kcal/mol, whereas other possible bioactive phytochemicals including melongoside O and melongoside G had the best docking values of −9.01 and −8.11 Kcal/mol, respectively.

### 3.2. Visualization of Post-Docking Protein-Ligand Interactions

**Figure 1:**
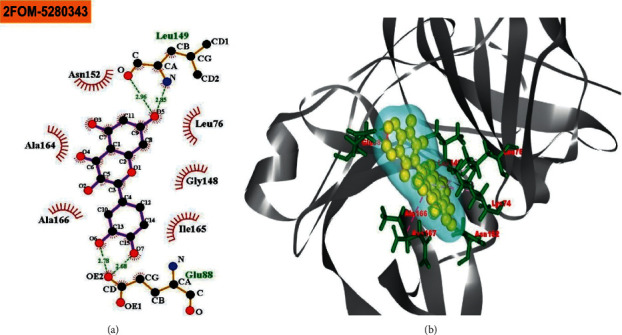
The interaction of the control compound quercetin 5280343 (pubchem CID) with the NS2B-NS3 protease of Dengue 2 (DENV-2) is displayed. The left side represents the 3D complex, while the right side represents the 2D complex of the protein-ligand interaction.

**Figure 2:**
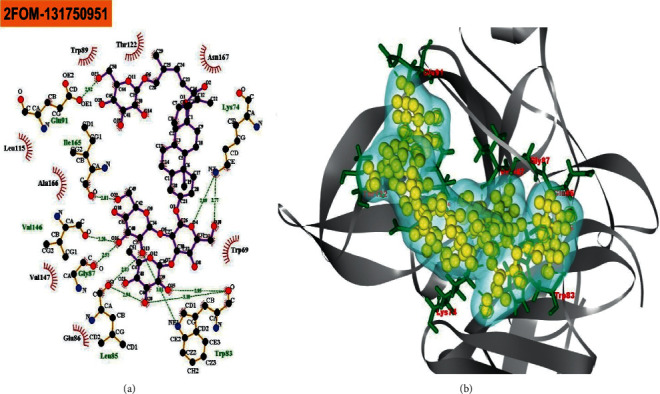
The interaction of the ligand compound melongoside P 131750951 (pubchem CID) with the NS2B-NS3 protease of dengue 2 (DENV-2) is displayed. The left side represents the 3D complex, while the right side represents the 2D complex of the protein-ligand interaction.

**Figure 3:**
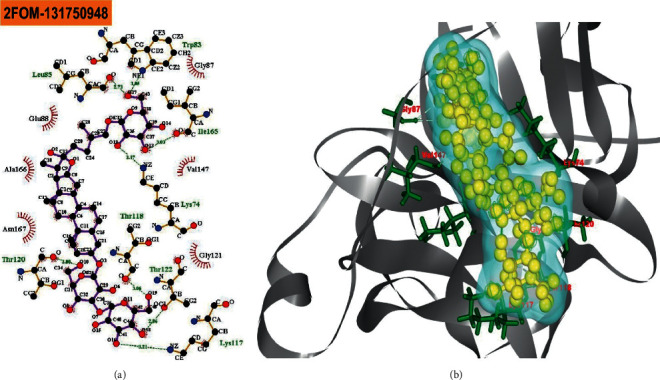
The interaction of the ligand compound melongoside O 131750948 (pubchem CID) with the NS2B-NS3 protease of dengue 2 (DENV-2) is displayed. The left side represents the 3D complex, while the right side represents the 2D complex of the protein-ligand interaction.

**Figure 4:**
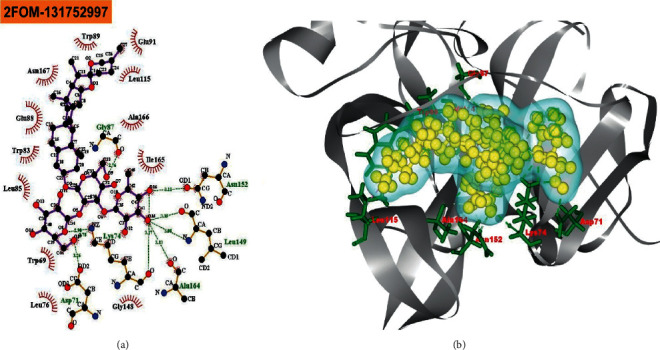
The interaction of the ligand compound melongoside G 131752997 (pubchem CID) with the NS2B-NS3 protease of dengue 2 (DENV-2) is displayed. The left side represents the 3D complex, while the right side represents the 2D complex of the protein-ligand interaction.

The BIOVIA Discovery Studio Visualizer and Ligplot + Version 2.2 tools have been used to investigate the interactions between the four ligands that were specified and the target protein in this study. Ligplot + version 2.2 was performed to calculate the interactions (which were predominantly hydrophobic and noncovalent) for all the docked complexes as shown in (Table 1) and (Figures 1–4).

The reference drug quercetin (CID-5280343) interacted with the NS2B-NS3 protease of Dengue 2 and showed four hydrogen bonds (with Leu149 (2.96 Å), Leu149 (2.85 Å), Glu88 (2.78 Å), and Glu88 (2.68 Å)), and six hydrophobic bonds (with Asn152, Ala164, Ala166, Leu76, Gly148, and Ile165) (Figure 1). The “NS2B-NS3 protease-melongoside P” complex was supported by eleven hydrogen bonds (Glu91 (2.91 Å), Ile165 (2.83 Å), Lys74 (2.89 Å), Lys74 (2.77 Å), Trp83 (2.95 Å), Trp83 (3.18 Å), Trp83 (3.01 Å), Leu85 (2.83 Å), Leu85 (2.94 Å), Gly87 (2.53 Å), and Val146 (3.28 Å)) and eight hydrophobic bonds (Trp89, Thr122, Asn167, Leu115, Ala166, Val147, Glu86, and Trp69) (Figure 2), whereas melongoside O interaction with DENV-2 NS2B-NS3 protease was supported by eight hydrogen bonds (Trp83 (3.05 Å), Leu85 (2.73 Å), Ile165 (3.03 Å), Lys74 (3.17 Å), Thr118 (3.06 Å), Thr122 (2.86 Å), Lys117 (3.21 Å), and Thr120 (3.08 Å)) and six hydrophobic bonds (Glu88, Ala166, Asn167, Gly121, Val147, and Gly87) (Figure 3).

Eight hydrogen bonds (Gly87 (2.76 Å), Asn152 (3.11 Å), Leu149 (3.35 Å), Leu149 (3.08 Å), Ala164 (3.13 Å), Lys74 (2.90 Å), Lys74 (3.17 Å), and Asp71 (3.26 Å)) and twelve hydrophobic connections (Trp89, Glu91, Leu115, Ala166, Ile165, Gly148, Leu76, Trp69, Leu85, Trp83, Glu88, and Asn167) were shown by melongoside G with the NS2B-NS3 protease of DENV-2.

### 3.3. Molecular Dynamic Simulation

Analysis of the biomolecular interactions and the analysis of the interface between the arrangement and activity of proteins can aid in the development of new drugs, and the performance data from the dynamic trajectory analysis named the molecular dynamic simulation (MDS) [24, 25] are used to analyze the stability and the intermolecular interactions of a protein-ligand complex in real-time. When a sophisticated system is subjected to an artificial environment, this technique can also be used to determine its conformational change. To better understand the conformational changes of the protein in complex, a 200 ns MD simulation of the protein in connection with the specific ligand was performed in this study. The terminal snapshots from the MDS trajectories were used to examine the intermolecular behavior initially.

### 3.4. RMSD Analysis

With a range of 1–3 Å, the average change in the root means square deviation (RMSD) of the protein-ligand interaction is acceptable. If the RMSD number is larger than 1–3, it means the protein structure has changed significantly. An MD simulation (200 ns) was run and the associated RMSD value was determined to assess the conformational change of the desired protein in the complex with the four ligand compounds, namely, CID-5280343, CID-131750951, CID-131750948, and CID-131752997.

**Figure 5:**
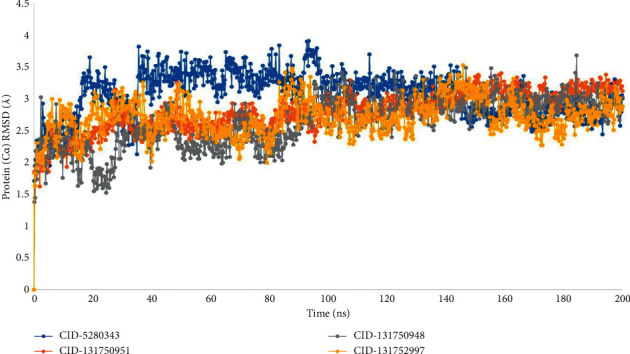
The RMSD values for the dengue 2 (DENV-2) NS2B-NS3 protease in complex with the four ligand compounds recovered from the complex system's C atoms, where the selected four ligand compounds to compound CID-5280343, CID-131750951, CID-131750948, and CID-131752997 in the complex with the protein are represented by blue, orange, grey, and yellow color, respectively.

The average root means square deviation (RMSD) for the ligand compounds CID-131750951, CID-131750948, and CID-131752997 was 1.5–3. Another ligand molecule, CID-5280343, was shown to have a potent RMSD value in the 1.5–3.5 range. The compound's value change shows very little fluctuation, which is within the permitted range, indicating that the protein-ligand complex structure represented in (Figure 5) is conformationally stable.

### 3.5. RMSF Analysis

**Figure 6:**
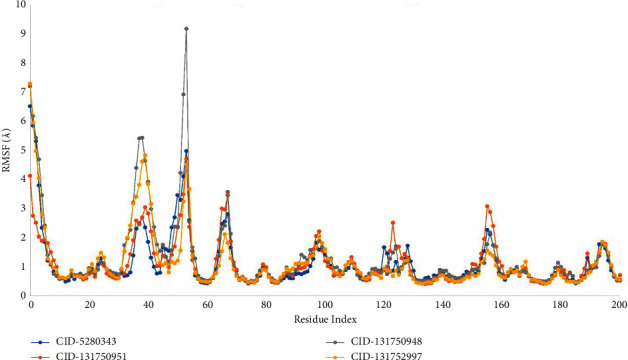
The RMSF values for the protein C atoms in docked protein-ligand complexes were calculated, where blue, orange, grey, and yellow represent the selected four ligand compounds in contact with the protein CID-5280343, CID-131750951, CID-131750948, and CID-131752997, respectively.

When the specific ligand chemicals interact with certain residues, the root mean square fluctuation (RMSF) can help characterize and determine the local alterations that occur within the protein chain. As a result, the RMSF values of the compounds CID-5280343, CID-131750951, CID-131750948, and CID-131752997 in complex with the Dengue 2 (DENV-2) NS2B-NS3 protease were calculated in order to investigate the change in protein structural flexibility caused by the attachment of specific ligand compounds to a specific residual position as shown in (Figure 6).

The most rigid secondary structural components, such as alpha-helices and beta-strands, were shown to have a minimum observation rate of 5 to 290 amino acid residues. The majority of the variation is seen at the beginning and at the end of the protein due to the presence of the N- and C-terminal domains. As a result, the displacement of an individual atom in the simulated environment has a low fluctuation probability for the four ligand complexes investigated.

### 3.6. The Radius of Gyration (Rg) Analysis

The arrangement of its atoms across its axis defines the radius of gyration (Rg) of a protein-ligand interaction system. The calculation of Rg is one of the most important indicators to look for when anticipating a macromolecule's structural functioning since it shows variations in complex compactness over time.

**Figure 7:**
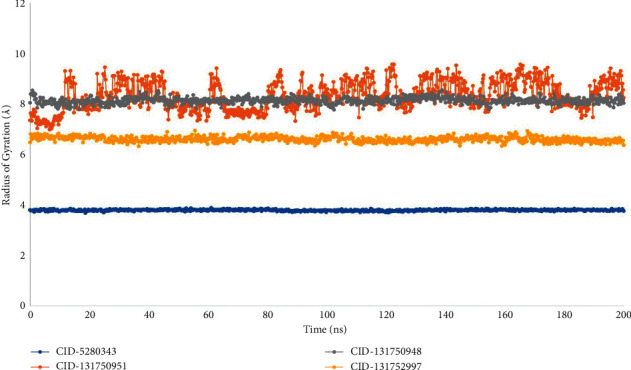
The radius of gyration (Rg) of the protein-ligand interaction was calculated using a 200 ns simulation, where blue, orange, grey, and yellow represent the selected three ligand compounds in contact with the protein CID-5280343, CID-131750951, CID-131750948, and CID-131752997, respectively.

As shown in (Figure 7), the stability of CID-5280343, CID-131750951, CID-131750948, and CID-131752997 in interaction with the target protein was studied in terms of Rg throughout a 200 ns simulation duration. The average Rg values for the compounds' CID-5280343, CID-131750951, CID-131750948, and CID-131752997 were 3.9, 8.1, 8, and 6.75, respectively, indicating that the protein's binding site does not undergo major structural changes when the ligand compounds are bound.

### 3.7. Analysis of SASA, MolSA, and PSA

The amount of solvent-accessible surface area (SASA) regulates the arrangement and activities of biological macromolecules. In most cases, amino acid residues on a protein's surface serve as active sites and/or interact with other molecules and ligands, allowing researchers to better understand a molecule's solvent-like behavior (hydrophilic or hydrophobic) and protein-ligand interaction components.

**Figure 8:**
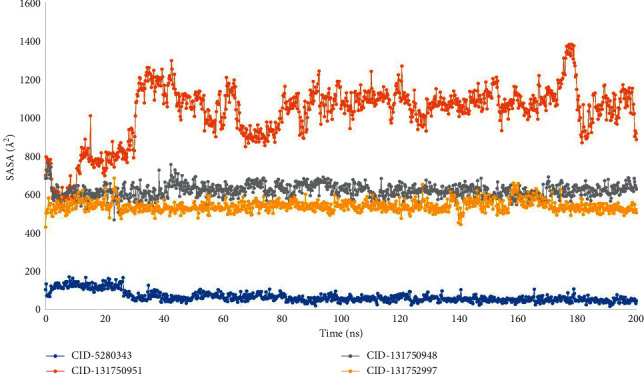
The 200 ns simulated interaction diagram was used to calculate the solvent-accessible surface area (SASA) of the protein-ligand interaction complexes, where blue, orange, grey, and yellow represent the selected four ligand compounds in contact with the protein CID-5280343, CID-131750951, CID-131750948, and CID-131752997, respectively.

The SASA values for the protein complexes with CID-5280343, CID-131750951, CID-131750948, and CID-131752997 were calculated and are presented in (Figure 8). The SASA values for the three compounds CID-131750951, CID-131750948, and CID-131752997 were 500 to 1200 A^2^ on average, and 100 A^2^ for CID-5280343, showing that in the complex systems, an amino acid residue was exposed to a high amount of the selected ligand molecules.

**Figure 9:**
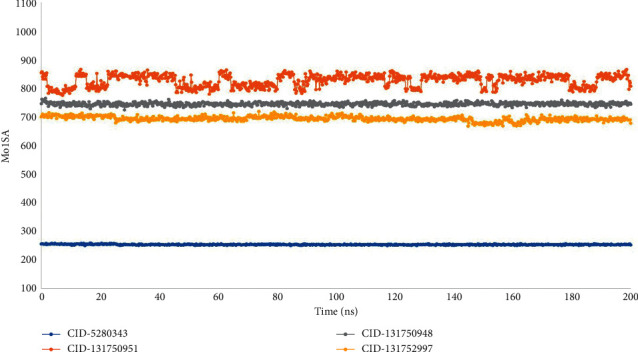
From the 200 ns simulated interaction diagram, the molecular surface area (MolSA) of the protein-ligand interaction compounds was estimated, where blue, orange, grey, and yellow represent the selected three ligand compounds in contact with the protein CID-5280343, CID-131750951, CID-131750948, and CID-131752997, respectively.

**Figure 10:**
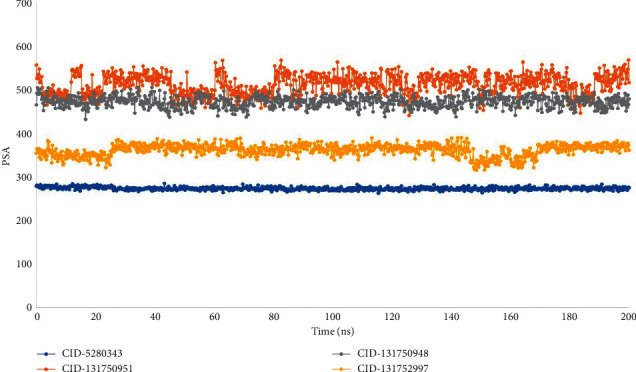
From the 100 ns simulated interaction diagram, the polar surface area (PSA) of the protein-ligand interaction compounds was estimated, where blue, orange, grey, and yellow colors represent the selected three ligand compounds in contact with the protein, CID-5280343, CID-131750951, CID-131750948, and CID-131752997 respectively.

The molecular surface area (MolSA) is the same as the van der Waals surface area calculated with a probe radius of 1.4. All of the ligand complexes CID-5280343, CID-131750951, CID-131750948, and CID-131752997 had the typical van der Waals surface area in our in-silico investigation (Figure 9). Furthermore, only oxygen and nitrogen atoms contribute to a molecule's polar surface area (PSA). With the targeted protein, all the ligand molecules CID-6474309, CID-5280805, and CID-442658 had a high PSA value (Figure 10).

### 3.8. Analysis of Intramolecular Bonds

The complex structure of a protein with the selected ligands and their intermolecular interactions was investigated for a 200 ns simulation time using the simulation interactions diagram (SID).

**Figure 11:**
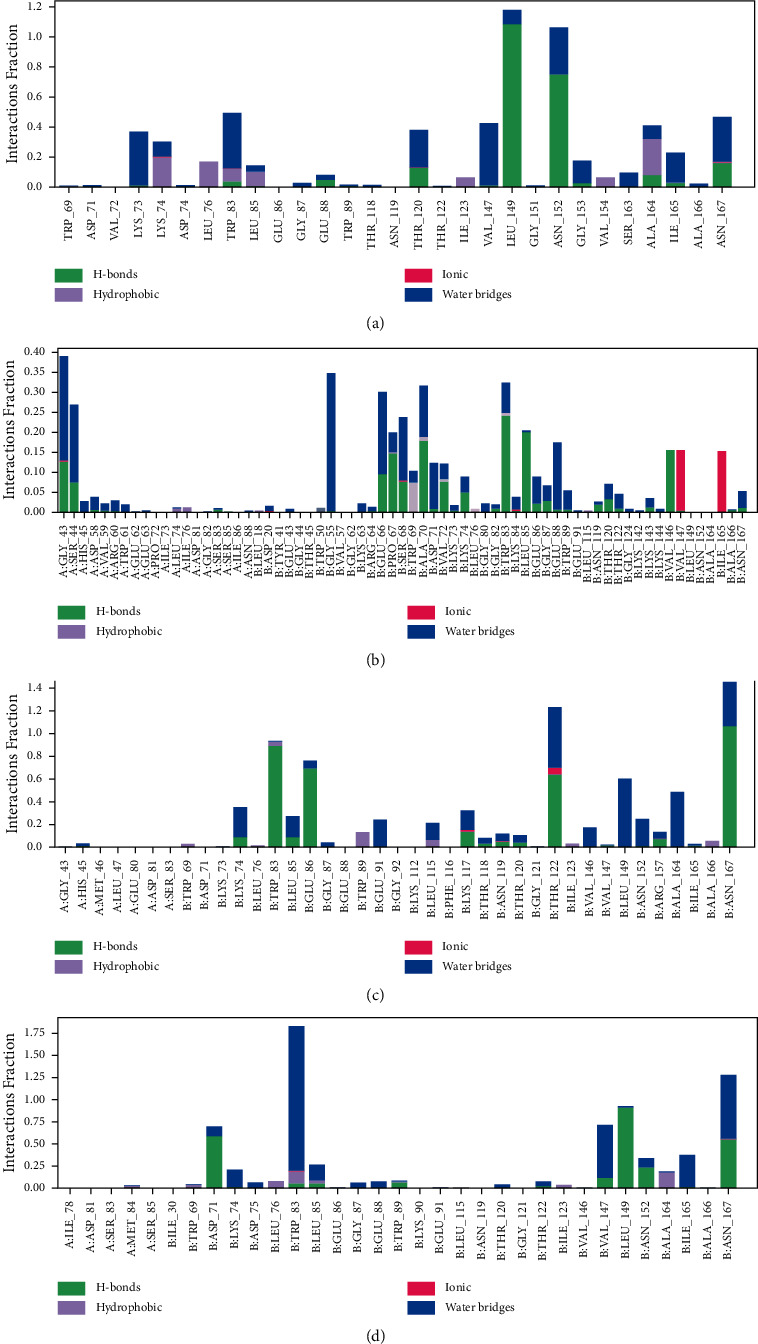
During the 200 ns simulation, the interactions between proteins and ligands were discovered, as seen in the stacked bar charts. This section depicts the interaction of four different compounds, where the selected four ligands (a) CID-5280343, (b) CID-131750951, (c) CID-131750948, and (d) CID-442658 are in contact with the protein.

The hydrogen bond, the ionic bond, the water bridge bond, and the noncovalent interactions bond (hydrophobic bond) defines the interaction between the protein and the designated ligands, which were evaluated and displayed for CID-5280343, CID-131750951, CID-131750948, and CID-131752997 (Figure 11). All compounds formed several connections via hydrogen, ionic, water bridge, and hydrophobic bonding over the 200 ns simulation time and retained these contacts until the simulation concluded, assisting in the establishment of a stable binding with the targeted protein.

## 4. Discussion

In recent years, the *in silico* drug design method has gained widespread acceptance due to its ability to accelerate quality drug development by evaluating the results of pharmacophore screening, molecular docking, analysis of postdocking interaction, molecular dynamic simulation (MDS), and prediction of noble drug compounds against a wide range of diseases in a computer-simulated environment [23]. Three out of the four phytochemicals utilized in this study were functionally selected as the active compounds, while another one was employed as a control.

Molecular docking is a technique for determining how two or more molecules will interact in the presence of the highest compositional confirmation and the lowest binding affinity conceivable [26, 27]. Drug candidates that delivered the most significant and stable score were selected using the Maestro application (Schrödinger Release 2021-2: Maestro, Schrödinger, LLC, New York, NY, 2020-3.), which utilizes molecular docking to assign a score. The molecular docking study for the selected three natural bioactive compounds and the control drug with the NS2B-NS3 protease of Dengue 2 (DENV-2), PDB ID-2FOM reported the docking affinity of the control drug quercetin (Pubchem CID-5280343) was −8.2 Kcal/mol. Among the selected natural bioactive compounds, melongoside P (Pubchem CID-131750951) possessed the best binding affinity of −9.5, in which melongoside O (Pubchem CID-131750948) was −9.01, melongoside G (Pubchem CID-131752997) was −8.11, and all the docking affinity results with the chemical names of the ligands are depicted in Table 1. In the following step, Ligplot + (Version 2.2), an excellent investigative tool that normally operates through the Java interface, was employed to investigate the 2D protein-ligand interaction scheme. Furthermore, the Discovery Studio Visualizer tool v19.1.0.18287 (BIOVIA), an effective visualizer tool for drug discovery, was used to represent the postdocking receptor-ligands interactions with their animated structures (Figures 1–4).

Our study was conducted with the Schrödinger package software (Desmond Application) to run 200 ns molecular dynamic simulation (MDS) with the selected physiological and physicochemical parameters. This simulation trajectory of the simulation tool has also been used to perfectly analyze the root mean square deviation (RMSD), root means square fluctuation (RMSF), the radius of gyration (Rg), hydrogen bond number, and solvent-accessible surface area (SASA) [28]. The root means square deviation (RMSD) of the selected NS2B-NS3 protease of Dengue 2 (DENV-2) protein's alpha carbon and backbone was used to evaluate the protein structure's reliability and identify conformational changes; the lower value indicates the most stable compounds. The RMSD values of less than 1.5 Å were typically indicative of greater consistency in docking since RMSD values over 1.5 Å typically indicate the average binding positions. In our study, the RMSD values of protein-ligand interactions were within an appropriate range, namely, the average mean values of 2.5 (the lowest value for the selected ligand compounds was approximately 0.8 and maximum value was 3), suggesting a better docking position and no disruption of the protein-ligand structure (Figure 5). Individuals can quantify the average protein fluctuations from such a reference location using the RMSF and the RMSF plots demonstrate how the average protein fluctuations represent fluctuations at the residue level. An illustration of the RMSF of the c-alpha atoms can be found in (Figure 6).

This conformational stability was determined by counting the complete number of intermolecular bonds generated among the macromolecules and their ligands, and the highest number of intramolecular bonds were determined as melongoside P and melongoside O, which are more stable in conformation than that of the control compound (Figure 11). The protein-ligands solvent-accessible surface area (SASA) was also determined using the simulation trajectories in order to determine the dimensional changes of the drug-like molecules along the simulation trajectory [29]. One of the greatest SASA values owes to the unstable structure, which contained hydrophobic amino acid residues close to the water molecule [30]. According to the SASA result from the MDS trajectory, melongoside P exhibited the highest SASA values, in which (−)-melongoside O, and melongoside G had the higher SASA values than the control drug, quercetin (Figure 8). In the graph of MolSA and PSA validation, all the ligands' compounds possessed more potential value than the control drug (Figures 9 and 10).

Additionally, Rg is a measurement of the distance between the portion of the center mass and the end portion of the protein, and it possesses how far that distance is. As a result, this metric quantifies the protein molecule's compactness and provides further information about the protein's folding properties [31]. Furthermore, a larger Rg value denotes slack packing, whereas a lower Rg value denotes compact packing [32]. The Rg values are summarized in Figure 7 and show that all the ligands with the protein showed standard compactness compared to the control drug. However, there are insufficient clinical research findings based on these ligand compounds to suggest it as a therapeutic option for Dengue virus treatment. Consequently, these biologically active phytocompounds can be considered a potential option for SARS-CoV-2 medication when they will have been validated for anti-Dengue virus activity in *in-vitro* and *in-vivo* research models.

## 5. Concluding Remarks

A mosquito-borne viral disease, dengue, has grown dramatically in recent years and become a global burden that affects most Asian and Latin American countries. There are no available specific drugs for dengue disease as well. Our present experiment that was evaluated by molecular docking and dynamics simulations techniques suggests further attention towards steroidal saponins (melongosides) as a DENV inhibitory agent, which if proven *In Vivo* and in clinical trials, can be a useful therapeutic target against at least DENV-2.

## Figures and Tables

**Table 1:** Molecular interactions between the chosen phytochemicals and the targeted receptor are tabulated in the docking score.

Compounds	Docking score (Kcal/mol)	Amino acid participation in bonding interaction	
		Interaction of the hydrogen bond	Interaction of the hydrophobic bond
Quercetin (reference) (CID-5280343)	−8.2	Leu149 (2.96 Å), Leu149 (2.85 Å), Glu88 (2.78 Å), Glu88 (2.68 Å)	Asn152, Ala164, Ala166, Leu76, Gly148, Ile165

Melongoside P (CID-131750951)	−9.5	Glu91 (2.91 Å), Ile165 (2.83 Å), Lys74 (2.89 Å), Lys74 (2.77 Å), Trp83 (2.95 Å), Trp83 (3.18 Å), Trp83 (3.01 Å), Leu85 (2.83 Å), Leu85 (2.94 Å), Gly87 (2.53 Å), Val146 (3.28 Å)	Trp89, Thr122, Asn167, Leu115, Ala166, Val147, Glu86, Trp69

Melongoside O (CID-131750948)	−9.01	Trp83 (3.05 Å), Leu85 (2.73 Å), Ile165 (3.03 Å), Lys74 (3.17 Å), Thr118 (3.06 Å), Thr122 (2.86 Å), Lys117 (3.21 Å), Thr120 (3.08 Å)	Glu88, Ala166, Asn167, Gly121, Val147, Gly87

Melongoside G (CID-131752997)	−8.11	Gly87 (2.76 Å), Asn152 (3.11 Å), Leu149 (3.35 Å), Leu149 (3.08 Å), Ala164 (3.13 Å), Lys74 (2.90 Å), Lys74 (3.17 Å), Asp71 (3.26 Å)	Trp89, Glu91, Leu115, Ala166, Ile165, Gly148, Leu76, Trp69, Leu85, Trp83, Glu88, Asn167

Melongoside N (CID-4483043)	−7.53	Leu149 (3.03 Å), Lys74 (3.08 Å), Asn152 (3.27 Å), Asn152 (2.73 Å), Lys117 (2.94 Å), Glu91 (2.89 Å)	Gly148, Leu76, Ile165, Ala166, Trp89, Asn167, Leu115, Glu88, Ala164

Melongoside F (CID-192242)	−6.67	Lys74 (3.08), Ile165 (3.24), Asn152 (2.85)	Glu88, Leu85, Asn167, Leu76, Gly148, Ala164, Ala166, Trp83

Melongoside H (CID-3826176)	−5.00	Ile165 (2.79), Trp83 (2.89), Leu85 (3.17), Lys 74 (3.07)	Leu115, Gly91, Glu88, Val147, Gly87, Lys90, Gly148

Melongoside B (CID-11827970)	−4.63	Asn152 (2.98), Ala164 (3.27)	Glu88, Lys90, Trp89, Ile165, Leu76, Gly148, Trp83, Lys74, Ala166, Asn167

## Data Availability

All the data used to support the findings of this study are included within the article.

## Abbreviations

DENV: Dengue virus
MDS: Molecular dynamic simulation
NSPs: Nonstructural proteins
Rg: Radius of gyration
RMSD: Root-mean-square deviation
RMSF: Root-mean-square fluctuation
SASA: Solvent-accessible surface area.

## References

[1] Yung C.-F., Lee K.-S., Thein T.-L. (2015). Dengue serotype-specific differences in clinical manifestation, laboratory parameters and risk of severe disease in adults, Singapore. *American Journal of Tropical Medicine and Hygiene*.

[2] Mustafa M. S., Rasotgi V., Jain S., Gupta V. (2015). Discovery of fifth serotype of dengue virus (DENV-5): a new public health dilemma in dengue control. *Medical Journal Armed Forces India*.

[3] Cox J., Mota J., Sukupolvi-Petty S., Diamond M. S., Rico-Hesse R. (2012). Mosquito bite delivery of dengue virus enhances immunogenicity and pathogenesis in humanized mice. *Journal of Virology*.

[4] (2022). Dengue and severe dengue. https://www.who.int/news-room/fact-sheets/detail/dengue-and-severe-dengue.

[5] Henchal E. A., Putnak J. R. (1990). The dengue viruses. *Clinical Microbiology Reviews*.

[6] Lescar J., Luo D., Xu T. (2008). Towards the design of antiviral inhibitors against flaviviruses: the case for the multifunctional NS3 protein from dengue virus as a target. *Antiviral Research*.

[7] Luo D., Vasudevan S. G., Lescar J. (2015). The flavivirus NS2B–NS3 protease–helicase as a target for antiviral drug development. *Antiviral Research*.

[8] Natarajan S. (2010). NS3 protease from flavivirus as a target for designing antiviral inhibitors against dengue virus. *Genetics and Molecular Biology*.

[9] Kiat T. S., Pippen R., Yusof R., Ibrahim H., Khalid N., Rahman N. A. (2006). Inhibitory activity of cyclohexenyl chalcone derivatives and flavonoids of fingerroot, *Boesenbergia rotunda* (L.), towards dengue-2 virus NS3 protease. *Bioorganic & Medicinal Chemistry Letters*.

[10] Raut R., Beesetti H., Tyagi P. (2015). A small molecule inhibitor of dengue virus type 2 protease inhibits the replication of all four dengue virus serotypes in cell culture. *Virology Journal*.

[11] Qaddir I., Majeed A., Hussain W., Mahmood S., Rasool N. (2020). An in silico investigation of phytochemicals as potential inhibitors against non-structural protein 1 from dengue virus 4. *Brazilian Journal of Pharmaceutical Sciences*.

[12] Mair C. E., Grienke U., Wilhelm A. (2018). Anti-influenza triterpene saponins from the bark of burkea africana. *Journal of Natural Products*.

[13] Zhou M., Xu M., Ma X.-X. (2012). Antiviral triterpenoid saponins from the roots of Ilex asprella. *Planta Medica*.

[14] Arthan D., Svasti J., Kittakoop P., Pittayakhachonwut D., Tanticharoen M., Thebtaranonth Y. (2002). Antiviral isoflavonoid sulfate and steroidal glycosides from the fruits of solanum torvum. *Phytochemistry*.

[15] Valadares Y. M., Brandão G. C., Kroon E. G., Souza Filho J. D., Oliveira A. B., Braga F. C. (2009). Antiviral activity of solanum paniculatum extract and constituents. *Zeitschrift für Naturforschung C*.

[16] Okubo K., Kudou S., Uchida T., Yoshiki Y., Yoshikoshi M., Tonomura M. (2010). ChemInform abstract: soybean saponin and isoflavonoids. Structure and antiviral activity against human immunodeficiency virus in vitro. *ChemInform*.

[17] Roner M. R., Tam K. I., Kiesling-Barrager M. (2010). Prevention of rotavirus infections *in vitro* with aqueous extracts of *Quillaja Saponaria* Molina. *Future Medicinal Chemistry*.

[18] Figueiredo G. G., Coronel O. A., Trabuco A. C. (2021). Steroidal saponins from the roots of solanum sisymbriifolium Lam. (solanaceae) have inhibitory activity against dengue virus and yellow fever virus. *Brazilian Journal of Medical and Biological Research*.

[19] Hariono M., Choi S. B., Roslim R. F. (2019). Thioguanine-based DENV-2 NS2B/NS3 protease inhibitors: virtual screening, synthesis, biological evaluation and molecular modelling. *PLoS One*.

[20] Senthilvel P., Lavanya P., Kumar K. M. (2013). Flavonoid from carica papaya inhibits NS2B-NS3 protease and prevents dengue 2 viral assembly. *Bioinformation*.

[21] Cerami E., Gao J., Dogrusoz U. (2012). The cBio cancer genomics portal: an open platform for exploring multidimensional cancer genomics data. *Cancer Discovery*.

[22] Hoffman J. M., Margolis K. G. (2020). Building community in the gut: a role for mucosal serotonin. *Nature Reviews Gastroenterology & Hepatology*.

[23] Bharadwaj S., Dubey A., Yadava U., Mishra S. K., Kang S. G., Dwivedi V. D. (2021). Exploration of natural compounds with anti-SARS-CoV-2 activity *via* inhibition of SARS-CoV-2 Mpro. *Briefings in Bioinformatics*.

[24] Subasri S., Viswanathan V., Kesharwani M., Velmurugan D. (2016). Phytochemical analysis, molecular docking and molecular dynamics simulations of selected phytoconstituents from four herbs as anti-dotes for snake bites. *Clinical Proteomics & Bioinformatics*.

[25] Aljahdali M. O., Molla M. H. R., Ahammad F. (2021). Compounds identified from marine mangrove plant (avicennia alba) as potential antiviral drug candidates against WDSV, an in-silico approach. *Marine Drugs*.

[26] Kapetanovic I. M. (2008). Computer-aided drug discovery and development (CADDD): in silico-chemico-biological approach. *Chemico-Biological Interactions*.

[27] Dey D., Paul P., Azad S. (2021). Molecular optimization, docking, and dynamic simulation profiling of selective aromatic phytochemical ligands in blocking the SARS-CoV-2 S protein attachment to ACE2 receptor: an in silico approach of targeted drug designing. *Journal of Advanced Veterinary and Animal Research*.

[28] Krupanidhi S., Abraham Peele K., Venkateswarulu T. C. (2021). Screening of phytochemical compounds of *Tinospora cordifolia* for their inhibitory activity on SARS-CoV-2: an *in silico* study. *Journal of Biomolecular Structure and Dynamics*.

[29] Elfiky A. A., Elshemey W. M. (2018). Molecular dynamics simulation revealed binding of nucleotide inhibitors to ZIKV polymerase over 444 nanoseconds. *Journal of Medical Virology*.

[30] Mahmud S., Rahman E., Nain Z. (2021). Computational discovery of plant-based inhibitors against human carbonic anhydrase IX and molecular dynamics simulation. *Journal of Biomolecular Structure and Dynamics*.

[31] Alamri M. A., Altharawi A., Alabbas A. B., Alossaimi M. A., Alqahtani S. M. (2020). Structure-based virtual screening and molecular dynamics of phytochemicals derived from Saudi medicinal plants to identify potential COVID-19 therapeutics. *Arabian Journal of Chemistry*.

[32] Kousar K., Majeed A., Yasmin F., Hussain W., Rasool N. (2020). Phytochemicals from selective plants have promising potential against SARS-CoV-2: investigation and corroboration through molecular docking, MD simulations, and quantum computations. *BioMed Research International*.

